# Supplementary material for: Performance bonuses and the quality of primary health care delivered by family health teams in Brazil: A difference-in-differences analysis
Source: PLoS Med. 2022 Jul 7;19(7):e1004033. doi: 10.1371/journal.pmed.1004033 (PMC9262241; doi:10.1371/journal.pmed.1004033)
Supplement: S1 Portuguese Abstract — (DOCX) [file pmed.1004033.s002.docx]

**Bônus baseados em desempenho e a qualidade da atenção primária à saúde prestada pelas equipes de saúde da família no Brasil: Uma análise de diferenças-em-diferenças**

# Resumo

**Introdução**: Os programas de pagamento por desempenho (P4P) têm sido amplamente implementados em todo o mundo para incentivar os provedores de cuidados de saúde a melhorar a qualidade dos cuidados. Apesar do apelo intuitivo, as evidências sobre a efetividade do P4P mostram resultados mistos, potencialmente devido a diferenças na forma como os esquemas são concebidos. Nós investigamos a variação na concepção do Programa Nacional de Melhoria do Acesso e Qualidade da Atenção Básica (PMAQ), entre os municípios brasileiros, com o intuito de examinar se a concessão de bônus por desempenho aos trabalhadores das equipes de saúde da família estava associada a mudanças na qualidade dos cuidados e se o tamanho desse bônus era importante.

**Métodos e resultados**: Para este estudo *quasi-experimental*, utilizamos uma abordagem de diferenças-em-diferenças, combinada com pareamento. Nós comparamos mudanças ao longo do tempo na qualidade dos cuidados prestados pelas equipes de saúde da família, entre municípios que optaram por utilizar parte ou a totalidade do recursos do PMAQ para fornecer incentivos financeiros baseados em desempenho aos trabalhadores das equipes de saúde (bônus) com municípios que investiram os recursos do PMAQ na forma tradicional de orçamento, baseado em insumos para o aprimoramento das unidades de saúde (sem-bônus). O desfecho principal foi a nota do PMAQ, um índice de qualidade dos cuidados numa escala de 0 a 100, baseado em várias centenas de indicadores (variando de 598 a 660) relacionados à prestação de cuidados de saúde. Fizemos o pareamento de um-para-um dos municípios com e sem bônus, considerando uma linha de base de características demográficas e econômicas. Na amostra pareada, utilizamos a regressão por mínimos quadrados ordinários para estimar a associação de qualquer bônus e do tamanho do bônus baseado nas mudanças ao longo do tempo (entre Novembro de 2011 e Outubro de 2015) na nota do PMAQ. Nós realizamos análises de subgrupos relativamente à renda da área local da equipe de saúde da família.

A amostra analítica pareada incluiu 2346 municípios (1173 municípios sem-bônus; 1173 municípios com bônus), contendo 10.275 equipes de saúde da família que participaram do PMAQ desde o início. Os municípios com bônus foram associados ao aumento de 4,6 (95% CI: 2,7 a 6,4; p <0,001) pontos percentuais na nota do PMAQ, em comparação com os municípios sem-bônus. A associação com a qualidade do cuidado aumentou com o tamanho do bônus: o grupo com maior bônus registou uma melhoria de 8,2 pontos percentuais (95% CI: 6,2 a 10,2; p<0,001), em comparação com o grupo controle. A análise do subgrupo mostrou que a melhoria observada no desempenho foi mais pronunciada nos dois quintis das localidades mais pobres.

As limitações do estudo incluem o potencial viés de confundimento de variáveis não mensuradas que variam ao longo do tempo, e o fato de a nota do PMAQ não ter sido validada como uma medida de qualidade de cuidados.

**Conclusão**: Os bônus de desempenho para os trabalhadores das equipes de saúde da família, em comparação com os orçamentos tradicionais baseados em insumos, foram associados a uma melhoria na qualidade dos cuidados.
