# Supplementary material for: Performance bonuses and the quality of primary health care delivered by family health teams in Brazil: A difference-in-differences analysis
Source: PLoS Med. 2022 Jul 7;19(7):e1004033. doi: 10.1371/journal.pmed.1004033 (PMC9262241; doi:10.1371/journal.pmed.1004033)
Supplement: S1 Table — (DOCX) [file pmed.1004033.s003.docx]

| **Variables** | | **Description** |  | **Source of data** |
| --- | --- | --- | --- | --- |
| **Outcome variable** | **Primary measure** |  |  |  |
|  | PMAQ score | Composite index of quality of care measured in each round of PMAQ at the level of family health team. Score ranges from from 0 (lowest possible score) to 100 (highest possible score). The outcome is the change in the PMAQ score between round 1 and round 3. |  | Ministry of Health, Brasilia |
| **Exposure variables** | **Incentive design variables** |  |  |  |
|  | Any bonus | Dummy variable that took the value of 1 if the municipality where the FHT is located passed at least some part of PMAQ funds as bonuses to frontline health workers. |  | Online survey of municipality health managers, administered by Ministry of Health |
|  | Size of bonus | Categorical variable indicating the size of the bonus as a percentage of average salaries of FHT workers |  | Online survey of municipality health managers, administered by Ministry of Health |
| **Control variables** | **Municipality level variables** |  |  |  |
|  | PMAQ funds in round 1 (in R$ 1,000) | Continuous variable indicating the average amount of PMAQ funds per FHT awarded to the municipality in the first round |  | PMAQ assessment, Ministry of Health, Brasilia (round 1, 2011) |
|  | GDP per capita (in R$ 1,000) | Continuous variable representing the municipal gross domestic product in 2010 |  | Municipality characteristics data, Brazilian Institute of Geography and Statistics, (2010) |
|  | Human development index | Composite index of life expectancy, income per capita and education measured at the level of municipality |  | Municipality characteristics data, Brazilian Institute of Geography and Statistics, (2010). Based on Census data. Available at: http://www.atlasbrasil.org.br/ |
|  | Gini index | A measure of income inequality and dispersion of wealth |  | Municipality characteristics data, Brazilian Institute of Geography and Statistics, (2010). Based on Census data. Available at: http://www.atlasbrasil.org.br/ |
|  | Total population | The population per each municipality |  | Municipality characteristics data, Brazilian Institute of Geography and Statistics, (2010). Based on Census data. Available at: http://www.atlasbrasil.org.br/ |
|  | Share of population urban | Percentage of population who lived in urban settings |  | Municipality characteristics data, Brazilian Institute of Geography and Statistics, (2010). Based on Census data. Available at: http://www.atlasbrasil.org.br/ |
|  | Share of population under 5 years | Percentage of population under the age of five |  | Municipality characteristics data, Brazilian Institute of Geography and Statistics, (2010). Based on Census data. Available at: http://www.atlasbrasil.org.br/ |
|  | Share of population over 60 years | Percentage of population over the age of sixty |  | Municipality characteristics data, Brazilian Institute of Geography and Statistics, (2010). Based on Census data. Available at: http://www.atlasbrasil.org.br/ |
|  | **Facility level variables** |  |  |  |
|  | Facility type | Categorical variable indicating the type of facility: health centre, health post, other |  | Health facility census, Ministry of health, Brasilia (2011) |
|  | Number of clinical staff | Continuous variable representing the number of staff working in a certain facility |  | Health facility census, Ministry of health, Brasilia (2011) |
|  | **Family health team level variables** |  |  |  |
|  | Income quintile | Categorical variable comprising five quintiles (equal sized groups) based on the average monthly income of households in each census area |  | Brazilian Census, Brazilian Institute of Geography and Statistics, (2010) |
