# Supplementary material for: Performance bonuses and the quality of primary health care delivered by family health teams in Brazil: A difference-in-differences analysis
Source: PLoS Med. 2022 Jul 7;19(7):e1004033. doi: 10.1371/journal.pmed.1004033 (PMC9262241; doi:10.1371/journal.pmed.1004033)
Supplement: S2 Table — The probit regression was run on municipality level data. CI, confidence interval; GDP, gross domestic product; PMAQ, National Programme for Improving Primary Care Access and Quality. (DOCX) [file pmed.1004033.s004.docx]

|  | Coefficient | 95% CI | P value |
| --- | --- | --- | --- |
| PMAQ score round 1 | 0.022 | 0.017 to 0.027 | <0.001 |
| GDP per capita | -0.002 | -0.005 to 0.002 | 0.365 |
| Human development index | -6.303 | -7.443 to -5.163 | <0.001 |
| Gini index | 2.057 | 1.241 to 2.874 | <0.001 |
| Total population | -0.006 | -0.027 to 0.015 | 0.548 |
| Share of population urban | 0.763 | 0.500 to 1.026 | <0.001 |
| Share of population under 5 years | -6.151 | -12.365 to 0.063 | 0.052 |
| Share of population over 60 years | 0.955 | -1.454 to 3.364 | 0.437 |
| R-squared | 0.059 |  |  |
| Number of municipalities | 3371 |  |  |
