# Supplementary material for: Performance bonuses and the quality of primary health care delivered by family health teams in Brazil: A difference-in-differences analysis
Source: PLoS Med. 2022 Jul 7;19(7):e1004033. doi: 10.1371/journal.pmed.1004033 (PMC9262241; doi:10.1371/journal.pmed.1004033)
Supplement: S3 Table — The standardised % bias is the % difference of the sample means in the treated and nontreated (full or matched) subsamples as a percentage of the square root of the average of the sample variances in the treated and nontreated groups. (DOCX) [file pmed.1004033.s005.docx]

| Variable | Match status | Treated mean | Control mean | Standardised % bias | % reduction in bias | t-test of difference in means | p value |
| --- | --- | --- | --- | --- | --- | --- | --- |
| PMAQ score round 1 | Unmatched | 60.1 | 59.0 | 11.7 |  | 3.34 | 0.001 |
|  | Matched | 59.7 | 59.6 | 1.5 | 87.1 | 0.37 | 0.711 |
| GDP per capita | Unmatched | 10.9 | 14.1 | -23.0 |  | -6.52 | <0.001 |
|  | Matched | 12.0 | 11.9 | 0.8 | 96.7 | 0.20 | 0.840 |
| Human development index | Unmatched | 0.647 | 0.676 | -42.9 |  | -12.38 | <0.001 |
|  | Matched | 0.659 | 0.657 | 2.5 | 94.1 | 0.60 | 0.546 |
| Gini index | Unmatched | 0.511 | 0.494 | 26.2 |  | 7.46 | <0.001 |
|  | Matched | 0.505 | 0.507 | -2.9 | 89.0 | -0.70 | 0.484 |
| Total population | Unmatched | 0.351 | 0.471 | -5.0 |  | -1.37 | 0.172 |
|  | Matched | 0.387 | 0.494 | -4.5 | 11.3 | -0.90 | 0.371 |
| Share of population urban | Unmatched | 0.637 | 0.663 | -11.7 |  | -3.36 | 0.001 |
|  | Matched | 0.649 | 0.646 | 1.5 | 87.2 | 0.36 | 0.717 |
| Share of population under 5 years | Unmatched | 0.074 | 0.071 | 22.4 |  | 6.37 | <0.001 |
|  | Matched | 0.073 | 0.073 | -0.7 | 96.8 | -0.17 | 0.869 |
| Share of population over 60 years | Unmatched | 0.122 | 0.123 | -4.0 |  | -1.15 | 0.251 |
|  | Matched | 0.122 | 0.123 | -1.6 | 60.7 | -0.38 | 0.706 |
