# Supplementary material for: Performance bonuses and the quality of primary health care delivered by family health teams in Brazil: A difference-in-differences analysis
Source: PLoS Med. 2022 Jul 7;19(7):e1004033. doi: 10.1371/journal.pmed.1004033 (PMC9262241; doi:10.1371/journal.pmed.1004033)
Supplement: S4 Table — The table presents the income subgroup effects as the difference between subgroups (with the poorest group acting as the reference category). Rather than reporting the p-value on each subgroup effect, we report the p-value from a Wald test that these income subgroup coefficients are jointly equal to zero. CI, confidence interval; PMAQ, National Programme for Improving Primary Care Access and Quality. (DOCX) [file pmed.1004033.s006.docx]

|  | Unmatched sample | | Matched Sample | |
| --- | --- | --- | --- | --- |
|  | Coefficient  (95% CI) | P value of joint test | Coefficient  (95% CI) | P value of joint test |
| **PMAQ bonus** |  |  |  |  |
| Poorest (reference) |  | 0.0015 |  | 0.0017 |
| Poorer | -1.47 (-3.58 to 0.64) |  | -2.01 (-4.5 to 0.5) |  |
| Middle | -3.51 (-6.37 to -0.66) |  | -5.04 (-8.4 to -1.7) |  |
| Richer | -4.83 (-7.75 to -1.91) |  | -5.77 (-9.5 to -2.1) |  |
| Richest | -2.62 (-5.57 to 0.33) |  | -3.56 (-7.1 to -0.1) |  |
|  |  |  |  |  |
| **1 to 20% of salaries** |  |  |  |  |
| Poorest (reference) |  | 0.0357 |  | 0.0109 |
| Poorer | -2.1 (-4.6 to 0.4) |  | -2.03 (-5 to 1) |  |
| Middle | -7.05 (-12.8 to -1.3) |  | -8.35 (-14 to -2.7) |  |
| Richer | -6.43 (-12.4 to -0.5) |  | -8.76 (-15.9 to -1.6) |  |
| Richest | -5.04 (-9.8 to -0.3) |  | -6.67 (-11.9 to -1.5) |  |
| **21 to 50% of salaries** |  |  |  |  |
| Poorest (reference) |  | 0.0334 |  | 0.0258 |
| Poorer | -0.24 (-2.9 to 2.4) |  | -1.77 (-5.1 to 1.5) |  |
| Middle | -1.77 (-6.4 to 2.9) |  | -3.72 (-9.2 to 1.8) |  |
| Richer | -4.91 (-9.4 to -0.4) |  | -6.28 (-11.6 to -0.9) |  |
| Richest | -0.81 (-4.6 to 3) |  | -2.2 (-6.7 to 2.3) |  |
| **More than 50% of salaries** |  |  |  |  |
| Poorest (reference) |  | 0.1095 |  | 0.1651 |
| Poorer | -1.15 (-4.6 to 2.3) |  | -1.84 (-5.8 to 2.1) |  |
| Middle | -1.01 (-5.3 to 3.3) |  | -2.91 (-7.9 to 2.1) |  |
| Richer | -3.14 (-7.7 to 1.4) |  | -4.08 (-9.5 to 1.3) |  |
| Richest | 1.62 (-2.9 to 6.1) |  | 0.51 (-4.6 to 5.7) |  |
