# Supplementary material for: Performance bonuses and the quality of primary health care delivered by family health teams in Brazil: A difference-in-differences analysis
Source: PLoS Med. 2022 Jul 7;19(7):e1004033. doi: 10.1371/journal.pmed.1004033 (PMC9262241; doi:10.1371/journal.pmed.1004033)
Supplement: S5 Table — The dependent variable is the change in the structural quality of care score, which is an index of quality between 0 and 100. The reference groups are as follows: for PMAQ bonus is nonbonus municipalities; for PMAQ bonus size is nonbonus municipalities; for local area is poorest; and for health centre is health post and others. CI, confidence interval; FHT, family health team; GDP, gross domestic product; PMAQ, National Programme for Improving Primary Care Access and Quality. (DOCX) [file pmed.1004033.s007.docx]

|  | Full (unmatched) sample | | | | |  | Matched sample | | | | |
| --- | --- | --- | --- | --- | --- | --- | --- | --- | --- | --- | --- |
|  | Any bonus to family health teams | |  | Size of bonuses | |  | Any bonus to family health teams | |  | Size of bonuses | |
|  | Coefficient  (95% CI) | P value |  | Coefficient  (95% CI) | P value |  | Coefficient  (95% CI) | P value |  | Coefficient  (95% CI) | P value |
| **PMAQ bonus** |  |  |  |  |  |  |  |  |  |  |  |
| Municipalities giving bonuses | 3.4 (1.9 to 5.0) | <0.001 |  |  |  |  | 3.3 (1.2 to 5.4) | 0.0022 |  |  |  |
| **PMAQ bonus size** |  |  |  |  |  |  |  |  |  |  |  |
| 1 to 20% of salaries |  |  |  | 3.9 (2.0 to 5.9) | <0.001 |  |  |  |  | 3.4 (1.1 to 5.8) | 0.0041 |
| 21 to 50% of salaries |  |  |  | 5.6 (3.4 to 7.8) | <0.001 |  |  |  |  | 5.6 (3.0 to 8.1) | <0.001 |
| More than 50% of salaries |  |  |  | 6.5 (2.9 to 10.1) | <0.001 |  |  |  |  | 6.4 (2.2 to 10.6) | 0.0027 |
| **Local area** |  |  |  |  |  |  |  |  |  |  |  |
| Poorer | -2.5 (-3.9 to 1.2) | <0.001 |  | -2.2 (-3.8 to 0.7) | 0.0046 |  | -3.4 (-5.0 to 1.9) | <0.001 |  | -3.2 (-5.1 to 1.4) | <0.001 |
| Middle | -3.7 (-5.6 to 1.9) | <0.001 |  | -3.4 (-5.6 to 1.3) | 0.0019 |  | -4.3 (-6.6 to 1.9) | <0.001 |  | -4.0 (-6.8 to 1.3) | 0.004 |
| Richer | -1.8 (-3.7 to 0.1) | 0.0583 |  | -2.2 (-4.4 to 0.1) | 0.0434 |  | -2.4 (-4.7 to 0.2) | 0.0349 |  | -2.9 (-5.6 to 0.3) | 0.0308 |
| Richest | -0.8 (-2.8 to 1.2) | 0.447 |  | -1.1 (-3.3 to 1.1) | 0.3311 |  | -1.3 (-3.7 to 1.1) | 0.2895 |  | -1.9 (-4.7 to 1.0) | 0.1937 |
| **Health facility** |  |  |  |  |  |  |  |  |  |  |  |
| Health centre | -0.4 (-1.4 to 0.5) | 0.3801 |  | -0.3 (-1.3 to 0.8) | 0.6341 |  | -0.1 (-1.3 to 1.1) | 0.8681 |  | 0.3 (-1.0 to 1.6) | 0.6665 |
| Number of clinical staff | 0.0 (-0.1 to 0.1) | 0.4263 |  | 0.0 (-0.1 to 0.1) | 0.388 |  | 0.0 (-0.1 to 0.1) | 0.5123 |  | 0.0 (-0.1 to 0.1) | 0.4923 |
| **Municipality characteristics** |  |  |  |  |  |  |  |  |  |  |  |
| PMAQ funds in round 1 (in R$ 1,000) | -0.5 (-0.9 to to0.2) | 0.0045 |  | -0.4 (-0.9 to 0.0) | 0.0474 |  | -0.6 (-1.1 to 0.2) | 0.0063 |  | -0.6 (-1.1 to 0.0) | 0.0418 |
| GDP per capita (in R$ 1,000) | 0.0 (-0.1 to 0.1) | 0.8513 |  | 0.0 (-0.1 to 0.2) | 0.6817 |  | 0.1 (-0.1 to 0.2) | 0.5133 |  | 0.2 (-0.1 to 0.4) | 0.2927 |
| Human development index | 2.8 (-14.9 to 20.4) | 0.7592 |  | 6.7 (-14.7 to 28.0) | 0.5414 |  | -3.6 (-25.6 to 18.5) | 0.7516 |  | -12.7 (-43.8 to 18.5) | 0.4257 |
| Gini index | 27.8 (13.0 to 42.7) | <0.001 |  | 35.1 (20.0 to 50.2) | <0.001 |  | 24.5 (7.4 to 41.7) | 0.0051 |  | 34.8 (17.6 to 52.0) | <0.001 |
| Total population | -0.0 (-0.1 to 0.1) | 0.6548 |  | -0.0 (-0.1 to 0.0) | 0.3596 |  | -0.0 (-0.1 to 0.1) | 0.7525 |  | -0.0 (-0.2 to 0.1) | 0.3817 |
| Share of population urban | -4.6 (-8.2 to 1.0) | 0.0134 |  | -4.7 (-8.9 to 0.5) | 0.03 |  | -2.8 (-7.3 to 1.8) | 0.2328 |  | -1.0 (-6.5 to 4.5) | 0.7209 |
| Share of population under 5 years | -0.9 (-93.9 to 92.2) | 0.9855 |  | 1.5 (-100.9 to 104.0) | 0.9766 |  | 26.5 (-84.7 to 137.7) | 0.6405 |  | 17.8 (-107.9 to 143.4) | 0.7817 |
| Share of population over 60 years | -9.4 (-51.3 to 32.5) | 0.6601 |  | -0.0 (-49.8 to 49.8) | 0.9997 |  | -2.1 (-53.6 to 49.4) | 0.9364 |  | 11.5 (-52.1 to 75.2) | 0.7222 |
|  |  |  |  |  |  |  |  |  |  |  |  |
| N teams | 13,050 |  |  | 10,564 |  |  | 9,783 |  |  | 7,600 |  |
| N municipalities | 3,290 |  |  | 2,692 |  |  | 2,291 |  |  | 1790 |  |
| R-squared | 0.0388 |  |  | 0.052 |  |  | 0.0382 |  |  | 0.0558 |  |
