# Supplementary material for: Performance bonuses and the quality of primary health care delivered by family health teams in Brazil: A difference-in-differences analysis
Source: PLoS Med. 2022 Jul 7;19(7):e1004033. doi: 10.1371/journal.pmed.1004033 (PMC9262241; doi:10.1371/journal.pmed.1004033)
Supplement: S6 Table — Results are from a lagged dependent variable model based on the full, unmatched, panel of family health teams. The dependent variable is the PMAQ score in round 3. Regressions are at the level of family health teams, with standard errors clustered at the municipality level. The reference groups are as follows: for PMAQ bonus is nonbonus municipalities; for PMAQ bonus size is nonbonus municipalities; for local area is poorest; and for health centre is health post and others. CI, confidence interval; FHT, family health team; GDP, gross domestic product; PMAQ, National Programme for Improving Primary Care Access and Quality. (DOCX) [file pmed.1004033.s008.docx]

|  | Full (unmatched) sample | | | | |
| --- | --- | --- | --- | --- | --- |
|  | Any bonus to family health teams | |  | Size of bonuses | |
|  | Coefficient  (95% CI) | P value |  | Coefficient  (95% CI) | P value |
| **PMAQ bonus** |  |  |  |  |  |
| Municipalities giving bonuses | 4.8 (3.3 to 6.3) | <0.001 |  |  |  |
| **PMAQ bonus size** |  |  |  |  |  |
| 1 to 20% of salaries |  |  |  | 3.1 (0.3 to 6.0) | 0.0325 |
| 21 to 50% of salaries |  |  |  | 6.8 (5.0 to 8.6) | <0.001 |
| More than 50% of salaries |  |  |  | 8.0 (5.9 to 10.1) | <0.001 |
| **Family health team** |  |  |  |  |  |
| PMAQ score in round 1 | 0.1 (0.0 to 0.2) | <0.001 |  | 0.1 (0.0 to 0.2) | <0.001 |
| **Local area** |  |  |  |  |  |
| Poorer | -1.3 (-2.1 to -0.5) | 0.0011 |  | -1.2 (-2.1 to -0.4) | 0.0029 |
| Middle | -1.3 (-2.2 to -0.4) | 0.0042 |  | -1.2 (-2.2 to -0.2) | 0.0171 |
| Richer | -1.5 (-2.5 to -0.5) | 0.0044 |  | -1.6 (-2.6 to -0.5) | 0.0028 |
| Richest | -0.5 (-1.7 to 0.7) | 0.3995 |  | -1.0 (-2.1 to 0.2) | 0.0979 |
| **Health facility** |  |  |  |  |  |
| Health centre | 0.0 (-0.7 to 0.7) | 0.9455 |  | 0.3 (-0.5 to 1.1) | 0.4409 |
| Number of clinical staff | -0.0 (-0.1 to 0.0) | 0.4934 |  | -0.0 (-0.0 to 0.0) | 0.5537 |
| **Municipality characteristics** |  |  |  |  |  |
| PMAQ funds in round 1 (in R$ 1,000) | 1.0 (0.6 to 1.3) | <0.001 |  | 1.0 (0.7 to 1.3) | <0.001 |
| GDP per capita (in R$ 1,000) | -0.0 (-0.1 to 0.0) | 0.1371 |  | -0.0 (-0.1 to 0.0) | 0.3800 |
| Human development index | 4.4 (-9.4 to 18.1) | 0.5352 |  | 10.1 (-3.8 to 24.1) | 0.1543 |
| Gini index | -1.0 (-24.2 to 22.2) | 0.9320 |  | 11.7 (0.0 to 23.4) | 0.0499 |
| Total population | 0.0 (-0.0 to 0.1) | 0.0592 |  | 0.0 (-0.0 to 0.1) | 0.2598 |
| Share of population urban | -0.9 (-4.1 to 2.3) | 0.5699 |  | -1.8 (-5.2 to 1.7) | 0.3219 |
| Share of population under 5 years | 16.9 (-77.3 to 111.1) | 0.7247 |  | 5.8 (-72.0 to 83.6) | 0.8835 |
| Share of population over 60 years | 23.7 (-17.3 to 64.6) | 0.2578 |  | 30.1 (-15.5 to 75.8) | 0.1957 |
|  |  |  |  |  |  |
| N teams | 13,716 |  |  | 11,060 |  |
| N municipalities | 3,371 |  |  | 2,761 |  |
| R-squared | 0.1062 |  |  | 0.1406 |  |
