# Supplementary material for: Performance bonuses and the quality of primary health care delivered by family health teams in Brazil: A difference-in-differences analysis
Source: PLoS Med. 2022 Jul 7;19(7):e1004033. doi: 10.1371/journal.pmed.1004033 (PMC9262241; doi:10.1371/journal.pmed.1004033)
Supplement: S7 Table — Each panel is a single robustness check, reporting results for the “any bonus” analysis and results for the “size of bonus” analysis. Panel A reports the results from the main analysis in the paper. Panel B is based on the unmatched sample and includes as additional controls: health care spending per capita and whether the political party of the municipality is the same as the national government. Panel C includes additional controls but is based on the matched sample. Panel D uses a smaller calliper of 0.001 in the matching procedure. Panel E uses a larger calliper of 0.2 in the matching procedure. Panel F reports results for size of bonus, including municipalities that gave a variable bonus amount to family health teams. CI, confidence interval. (DOCX) [file pmed.1004033.s009.docx]

| Any bonus to family health teams | | |  | Size of bonuses | | |
| --- | --- | --- | --- | --- | --- | --- |
| Exposure | Coefficient  (95% CI) | P value |  | Exposure | Coefficient  (95% CI) | P value |
| *Panel A. Matched sample, main results* | | |  |  |  |  |
| Any bonus | 4.6 (2.7 to 6.4) | <0.001 |  | 1 to 20% of salaries | 3.1 (-0.1 to 6.4) | 0.0609 |
|  |  |  |  | 21 to 50% of salaries | 6.4 (4.1 to 8.6) | <0.001 |
|  |  |  |  | More than 50% of salaries | 8.2 (6.2 to 10.2) | <0.001 |
| N teams | 10,275 |  |  |  | 7,938 |  |
| N municipalities | 2,346 |  |  |  | 1,836 |  |
| *Panel B. Unmatched sample, additional controls* | | |  |  |  |  |
| Any bonus | 4.4 (3.0 to 5.8) | <0.001 |  | 1 to 20% of salaries | 2.7 (-0.1 to 5.5) | 0.056 |
|  |  |  |  | 21 to 50% of salaries | 6.3 (4.4 to 8.3) | <0.001 |
|  |  |  |  | More than 50% of salaries | 7.3 (5.4 to 9.2) | <0.001 |
| N teams | 13,523 |  |  |  | 10,923 |  |
| N municipalities | 3,312 |  |  |  | 2,714 |  |
| *Panel C. Matched sample, additional controls* | | | | | | |
| Any bonus | 4.9 (3.3 to 6.5) | <0.001 |  | 1 to 20% of salaries | 3.4 (0.4 to 6.3) | 0.024 |
|  |  |  |  | 21 to 50% of salaries | 6.6 (4.4 to 8.9) | <0.001 |
|  |  |  |  | More than 50% of salaries | 7.9 (5.9 to 10.0) | <0.001 |
| N teams | 10,135 |  |  |  | 7,838 |  |
| N municipalities | 2,307 |  |  |  | 1,805 |  |
| *Panel D. Matched sample, matching caliper set at 0.001* | | | | | | |
| Any bonus | 4.2 (2.3 to 6.1) | <0.001 |  | 1 to 20% of salaries | 2.7 (-0.6 to 6.0) | 0.109 |
|  |  |  |  | 21 to 50% of salaries | 6.1 (3.6 to 8.6) | <0.001 |
|  |  |  |  | More than 50% of salaries | 8.1 (6.0 to 10.3) | <0.001 |
| N teams | 9,647 |  |  |  | 7,504 |  |
| N municipalities | 2,186 |  |  |  | 1,704 |  |
| *Panel E. Matched sample, matching caliper set at 0.2* | | | | | | |
| Any bonus | 4.8 (3.0 to 6.5) | <0.001 |  | 1 to 20% of salaries | 3.0 (-0.1 to 6.2) | 0.060 |
|  |  |  |  | 21 to 50% of salaries | 6.7 (4.6 to 8.8) | <0.001 |
|  |  |  |  | More than 50% of salaries | 8.2 (6.4 to 10.2) | <0.001 |
| N teams | 10,852 |  |  |  | 8,397 |  |
| N municipalities | 2,524 |  |  |  | 1,972 |  |
| *Panel F. Matched sample, inclusion of municipalities with variable bonus amount* | | | | | | |
| Any bonus | 4.6 (2.7 to 6.4) | <0.001 |  | 1 to 20% of salaries | 2.7 (-0.6 to 6.0) | 0.115 |
|  |  |  |  | 21 to 50% of salaries | 6.0 (3.9 to 8.2) | <0.001 |
|  |  |  |  | More than 50% of salaries | 8.4 (6.4 to 10.4) | <0.001 |
|  |  |  |  | Variable bonus amount | 3.4 (0.6 to 6.1) | 0.018 |
| N teams | 10,275 |  |  |  | 10,275 |  |
| N municipalities | 2,346 |  |  |  | 2,346 |  |
