# Supplementary figures and images for: Performance bonuses and the quality of primary health care delivered by family health teams in Brazil: A difference-in-differences analysis
Source: PLoS Med. 2022 Jul 7;19(7):e1004033. doi: 10.1371/journal.pmed.1004033 (PMC9262241; doi:10.1371/journal.pmed.1004033)

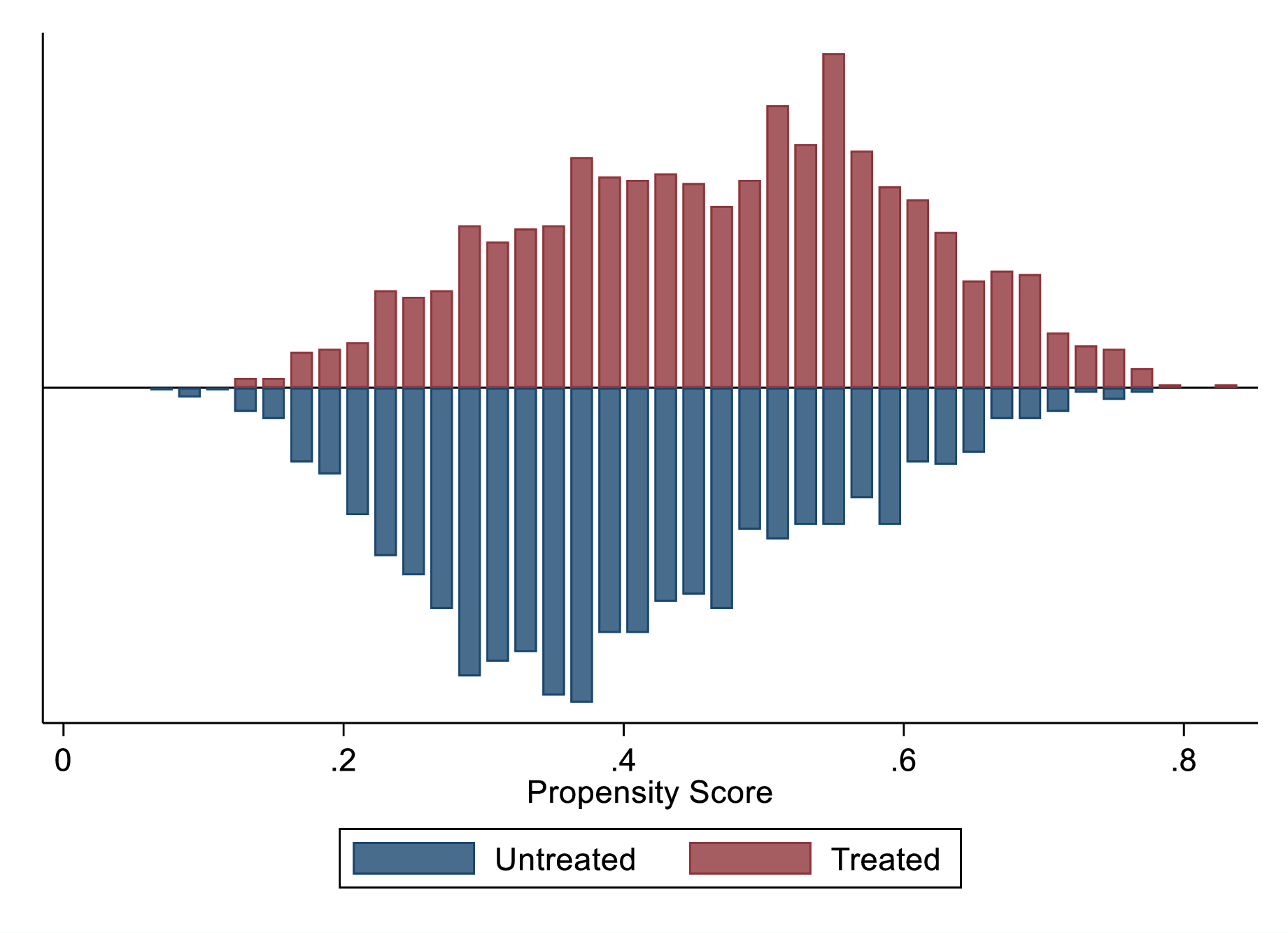

Supplement: S1 Fig — (TIF) [file pmed.1004033.s010.tif]

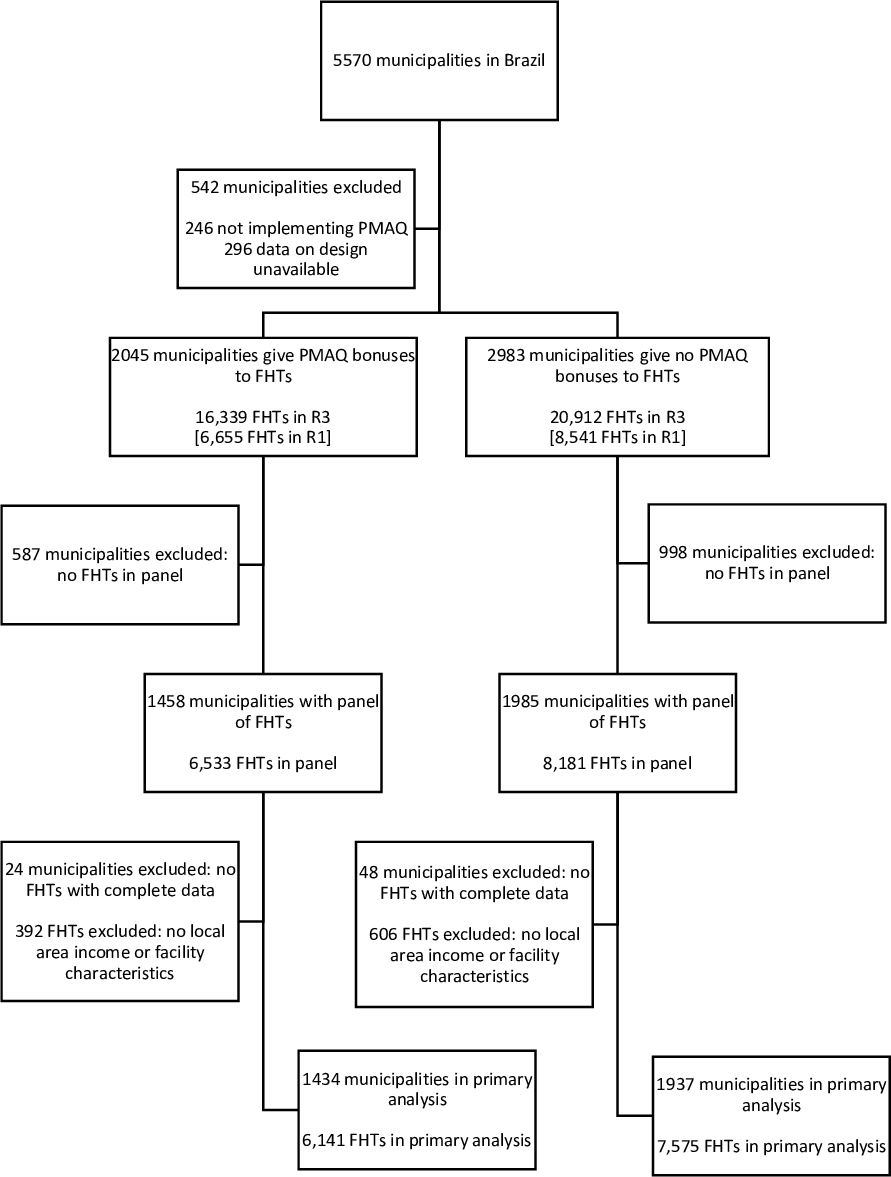

Supplement: S2 Fig — In the third step, some family health teams had no data on local area income because of missing geographical information to link them to the census area. (TIF) [file pmed.1004033.s011.tif]

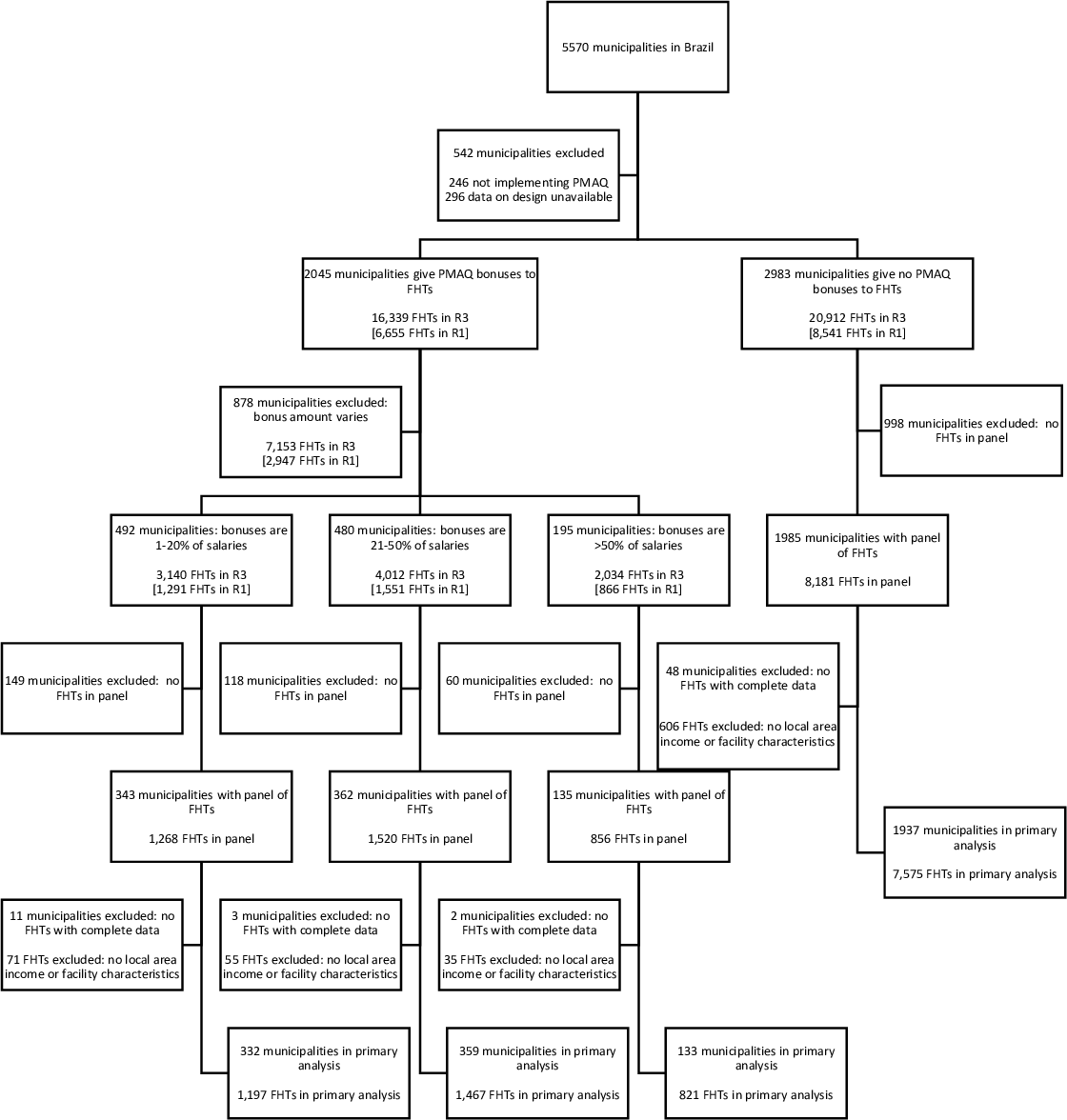

Supplement: S3 Fig — In the fourth step, some family health teams had no data on local area income because of missing geographical information to link them to the census area. (TIF) [file pmed.1004033.s012.tif]

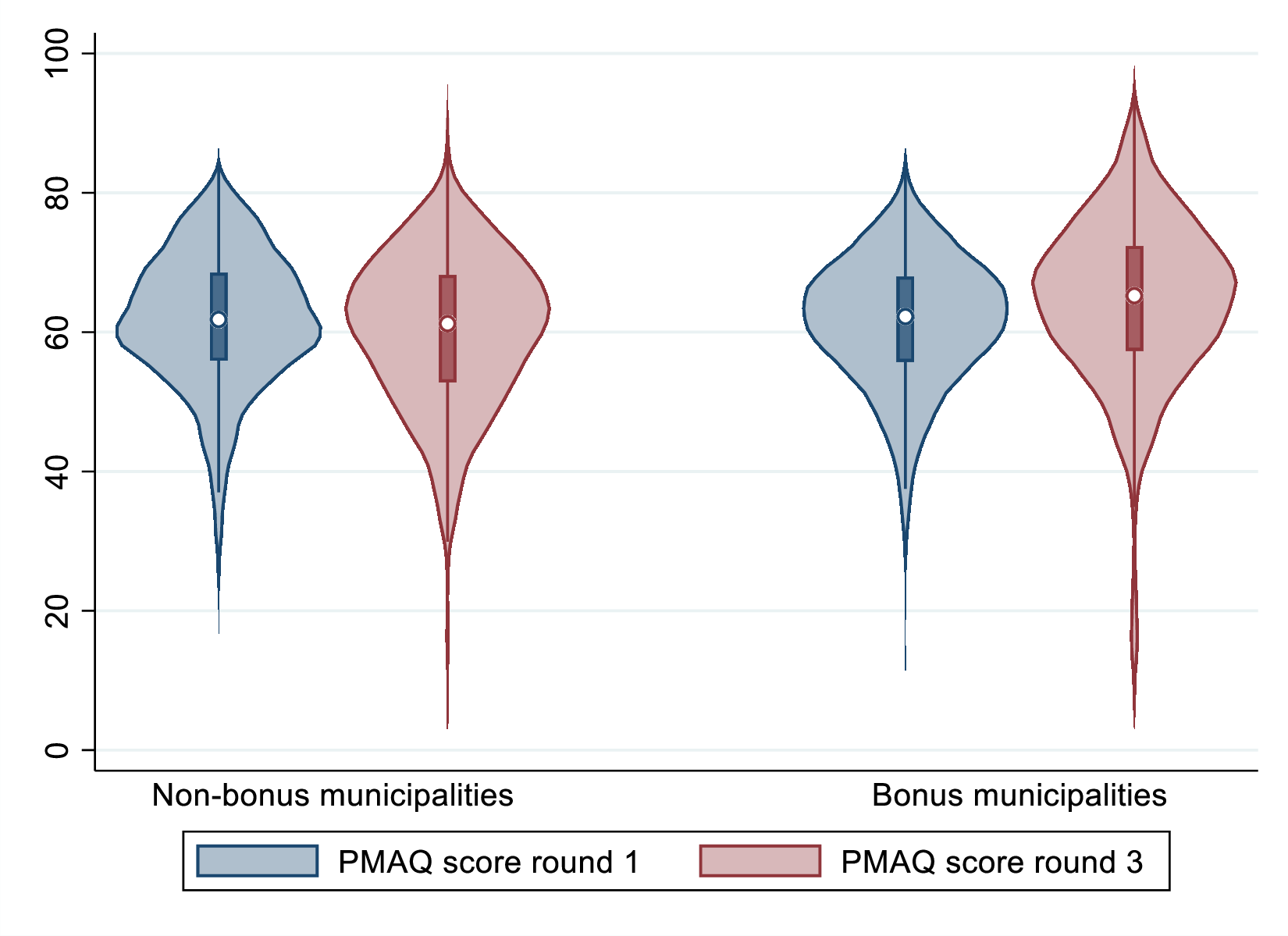

Supplement: S4 Fig — PMAQ, National Programme for Improving Primary Care Access and Quality. (TIF) [file pmed.1004033.s013.tif]

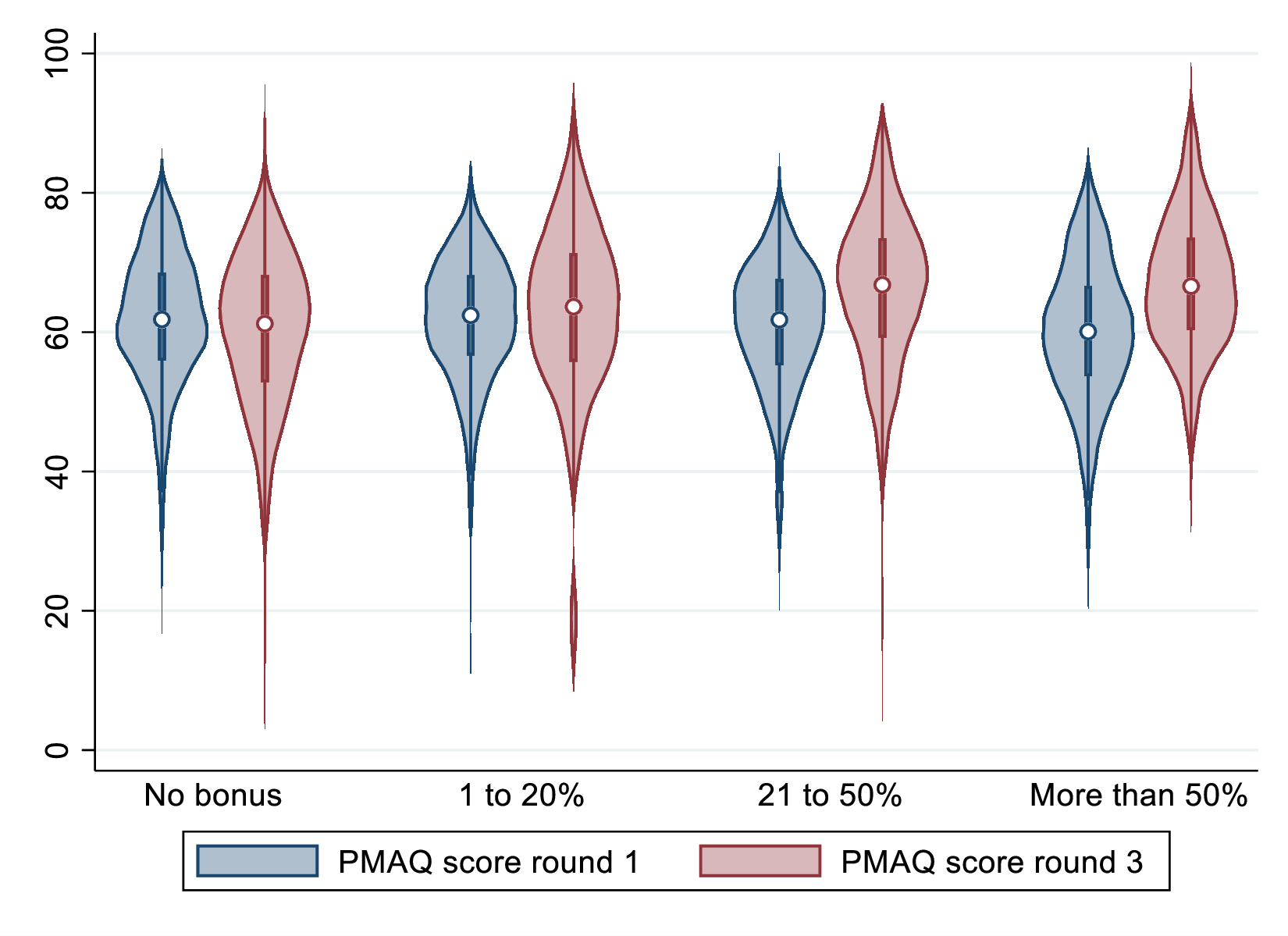

Supplement: S5 Fig — PMAQ, National Programme for Improving Primary Care Access and Quality. (TIF) [file pmed.1004033.s014.tif]
