# Supplementary material for: Performance bonuses and the quality of primary health care delivered by family health teams in Brazil: A difference-in-differences analysis
Source: PLoS Med. 2022 Jul 7;19(7):e1004033. doi: 10.1371/journal.pmed.1004033 (PMC9262241; doi:10.1371/journal.pmed.1004033)
Supplement: Attachment — Submitted filename: Responses.docx [file pmed.1004033.s018.docx]

We greatly appreciate the detailed comments provided by the reviewers, especially in the present climate when we are all facing additional work pressures. We have done our best to take the comments into account where appropriate. Please see our responses below in blue, which includes signposting of where we have made changes in manuscript.

## Editorial

1) Please consider identifying the study as a “a difference-in-differences” analysis. Please revise your title according to PLOS Medicine's style. Your title must be nondeclarative and not a question. It should begin with main concept if possible and avoid causal claims such as “Effect” or similar. The study design should remain in the subtitle (i.e., after a colon). Please consider, “Performance bonuses and the quality of primary care delivered by family health teams in Brazil: A difference-in differences analysis”

Thank you for your comment. We edited the title accordingly: Performance bonuses and the quality of primary health care delivered by family health teams in Brazil: A difference-in differences analysis

2) The Data Availability Statement (DAS) requires revision. For each data source used in your study, if the data are not freely available, please describe briefly the ethical, legal, or contractual restriction that prevents you from sharing it. Please also include an appropriate contact (web or email address) for inquiries (this cannot be a study author).

We have done this. Please note that we obtained the family health level data on the PMAQ score and the municipality manager data on local scheme design from an online survey from the Ministry of Health, covered by a collaborative agreement with them. We are not permitted to share these data. Both datasets can be requested from the Department for Family Health (Departamento de Saúde da Família – DESF) at the Ministry of Health of Brazil on the telephone number +55 61 33159044 or email [desf@saude.gov.br](mailto:desf@saude.gov.br).

We would appreciate discussing this further. One idea is for us to provide all the data and Stata analysis code except for the PMAQ score variables and the exposure variables (which we are not permitted to share). We can then replace the data we are unable to share with randomly generated values. This would mean others can run the analysis but would not be able to replicate our findings, unless they were to get access to the non-public data.

3) Abstract:

a) Please ensure that all numbers presented in the abstract are present and identical to numbers presented in the main manuscript text.

Thank you for your comment. The numbers have been updated.

b) Please include the important variables on which the municipalities were matched and any other adjustment variables.

We have done this. Please see our response to comment R3.2.

c) In the last sentence of the Abstract Methods and Findings section, please describe the main limitation(s) of the study's methodology.

We have done this. We have added to the abstract: The limitations of the study include unmeasured time-varying confounding, and the fact that the PMAQ score has not been validated as a measure of quality of care.

4) At this stage, we ask that you include a short, non-technical Author Summary of your research to make findings accessible to a wide audience that includes both scientists and non-scientists. The Author Summary should immediately follow the Abstract in your revised manuscript. This text is subject to editorial change and should be distinct from the scientific abstract. Please see our author guidelines for more information:

https://journals.plos.org/plosmedicine/s/revising-your-manuscript#loc-author-summary

We have done this.

5) Please conclude the Introduction with a clear description of the study hypothesis.

We have done this (Introduction, para 4).

6) Did your study have a prospective protocol or analysis plan? Please state this (either way) early in the Methods section.

There was no dedicated prospective study protocol or analysis plan for this paper. We now state this in the Methods (paragraph 6).

For information, the analysis in this paper is one of a number of outputs that fall under a project known as [EQUI-PMAQ](https://www.lshtm.ac.uk/research/centres-projects-groups/equipmaq). Our funding application for the project – a competitive grant application to the UK’s Medical Research Council and Brazil’s CONFAP – specified, as one of the key objectives, the research question addressed in this paper along with a brief description of the methods. We also developed a project study protocol that we submitted for ethics review in the UK and Brazil. Because this was an overarching study protocol, the methodological details for this specific paper were again brief.

a) If a prospective analysis plan (from your funding proposal, IRB or other ethics committee submission, study protocol, or other planning document written before analyzing the data) was used in designing the study, please include the relevant prospectively written document with your revised manuscript as a Supporting Information file to be published alongside your study, and cite it in the Methods section. A legend for this file should be included at the end of your manuscript.

b) If no such document exists, please make sure that the Methods section transparently describes when analyses were planned, and when/why any data-driven changes to analyses took place.

We have done this (Methods, paragraph 4).

c) In either case, changes in the analysis-- including those made in response to peer review comments-- should be identified as such in the Methods section of the paper, with rationale.

We did not make any changes to the analysis between the original plan and the submission of the paper. Our understanding is that we have not made many substantive changes to the analysis in our responses to the reviewer comments. One important change is the sensitivity analysis involving our structural quality of care index (see below). We now identify this analysis as new, based on the recommendations of a reviewer (Methods, Statistical Analysis, para 5). We did make a minor change to the estimation of the subgroup effects based on the comment by the statistical reviewer but we are not sure this warrants explanation.

7) Please clarify terms used such as “supply-side” and “input-based” so that it is clearer for readers who are not familiar with the health context in Brazil.

We have clarified the term in the Methods (paragraph 4). We also now use only “input-based budgets” to ensure consistency of language. Input-based budgets are to be contrasted with output-based payments. Paying on the basis of performance is an output-based payment. Paying for inputs (e.g. drugs, equipment, consumables, administration) is input-based financing.

8) Please ensure that the study is reported according to the STROBE guideline, and include the completed STROBE checklist as Supporting Information. Please add the following statement, or similar, to the Methods: "This study is reported as per the Strengthening the Reporting of Observational Studies in Epidemiology (STROBE) guideline (S1 Checklist)."

Done.

The STROBE guideline can be found here:

<http://www.equator-network.org/reporting-guidelines/strobe/>

9) When completing the STROBE checklist, please use section and paragraph numbers, rather than page numbers.

Done.

10) Could you please compare and comment on patient volumes in the bonus vs non-bonus municipalities and how this could be associated with improvement in quality of care? Is this incorporated in the PMAQ score?

We have no data on patient volumes and, to the best of our knowledge, they are not routinely available for family health teams over our study period. This is because the national health management information system was overhauled in the middle of our study period, causing disruption in routine data collection that affected municipalities differently.

The PMAQ score does indeed incorporate some, but not many, indicators of patient utilization, as reported by family health teams in the PMAQ external evaluations. Utilization indicators account for 0.9% of the indicators making up the PMAQ score. Please note we have data on the PMAQ score as constructed by the Ministry of Health – we do not construct the index ourselves from the underlying data. The precise indicators are listed in the government documents we now reference in the paper. See also our response to comment R1.2.

11) In the results text and tables, please quantify the main results with both 95% CIs and p values (where applicable).

Done.

12) Throughout the text, please remove language that implies causality, such as “effect” or similar. Refer to associations instead.

Done.

13) Please do not report P<0.0001; report as P < 0.001.

Done.

14) Please define all abbreviations used in tables and figures as footnotes. For example, PMAQ, CI, GDP, FHT

Done.

15) References:

a) Please select the PLOS Medicine reference style in your citation manager. In-text reference call outs should be presented as follows noting the absence of spaces within the square brackets: "... countries [1,2]."

Done.

b) Please update reference #43 or delete if they have not yet been published

We have removed this.

c) Please ensure that journal name abbreviations consistently match those found in the National Center for Biotechnology Information (NCBI) databases.

[https://journals.plos.org/plosmedicine/s/submission-guidelines#loc-references](https://protect-eu.mimecast.com/s/wYrWC8EVLUYNKXlFZnjgL?domain=journals.plos.org)

Done. We checked the journals in our list and corrected the abbreviation using the appropriate functionality in Endnote.

d) Ref #34 seems incomplete. Please include journal details or provide a weblink

This has been removed and we now reference what was reference 38.

e) Please provide access dates for all references with weblinks e.g., ref #42

Done.

16) Please remove the “Declaration of Interests” and “Funding” statements at the end of the main text. This information is captured in the metadata obtained in the submission form

Done.

17) To help us extend the reach of your research, please provide any Twitter handle(s) that would be appropriate to tag, including your own, your coauthors’, your institution, funder, or lab.

Comments from the Academic editor:

The methods and results quite cursory (one reviewer described as generic) so could these be made clearer? For example, what does "allocate funds on basis of performance" mean exactly--what % of the clinic funds was via the PMAQ bonus?

We have made revisions and now provide more detail in the Methods (as detailed in our responses to the reviewers below). We now explain what allocating funds on the basis of performance means. We do not know what % of clinic funds that were via the PMAQ funds. Brazil is highly decentralised. Federal funds are tracked down to the municipality level and we are not aware of data on expenditure below the municipality level. In our revisions, however, we do now report PMAQ funds as a % of federal funds for primary health care to give a sense of PMAQ’s importance from a financing perspective (Methods, paragraph 1).

A larger concern is about the definition of the outcome measure. What are we measuring exactly using 660 indicators? Which type of indicators predominate--access measures vs quality of care vs volume etc?

Reviewer 1 made the excellent point that different indicators went into the index at different time points, potentially rendering the outcome measure incomparable over time. The authors should clarify the extent of variation of the measure.

Moreover, a sensitivity analysis that takes a much smaller index indicating quality (versus the other components) that is stable over time would substantially strengthen the analysis

We agree with these comments and have made every effort to address them. We now give more details on the breakdown of the indicators into types of indicators (Methods, paragraph 2).

Importantly, we also now report results using a structural quality of care index that we constructed ourselves using common indicators across time. Please see our responses to R1.2 for much more information.

## Reviewer 1

| R1 | 1 | Reviewer #1: Thanks for the opportunity to review your manuscript. My role is as a statistical reviewer so my review concentrates on the study design, data, and the analysis. I have put general comments first, and followed these with queries relevant for a specific section of the manuscript (with a page/paragraph reference, page number starting from Abstract).  This study examines whether providing primary care provider team members with a bonus improves quality of care. The quality of care measurement is a composite of many indicators (>200) and used as a continuous variable. The health service areas (municipalities) could decide how to allocate extra funding, either as a bonus to the teams providing care or with centrally (by the municipality) funding of capacity or quality improvement schemes. The design included matching areas who provided bonuses to those that did not, and used a difference-in-difference approach from time-point 2 to the PMAQ score at the time-point 3.  I was pleased to see that allocation of PMAQ funding at round 1 was included in the propensity score matching - this was an obvious confounder as access to more resources is an obvious way to increase performance/quality. Probit regression was used to estimate the propensity scores - typically, logistic regression gets used here but Probit has potential advantages over logistic regression so this is fine. The analysis includes sensitivity analyses - an unmatched sample, with different matching options (i.e. calliper width), and options for the analysis (i.e. weighted by population and including variable bonuses). OLS is appropriate for the PMAQ as described - with this many indicators it should reliable able to be treated as a continuous variable. The sensitivity analyses were very similar to the main analysis. It is acknowledged that the exposure is based on self-reported data - I would be interested in seeing what the subject-matter reviewers think about potential for measurement error or bias here.  As we acknowledge in the paper, the exposure variable is based on responses from the municipality health manager to a survey administered as part of the “external evaluation” in the third round. While this person is the government official most informed about how the PMAQ money is used in a municipality, there is potential for measurement error but we do not have the data to make any further judgement. The other reviewers did not comment on this issue. |
| --- | --- | --- |
| R1 | 2 | One general issue I have is with the PMAQ. The authors are upfront that this is not a validated measurement of quality of clinical care - this again is one I'd be interested in seeing what the other reviewers think of this. From a data perspective, I am concerned that it seems that the indicators that make up the PMAQ at each time-point could be different. Is there any information that could be added to quantify how many component questions/indicators were dropped or added at each time-point in the study? I would not be concerned if it were only a very few that change, but if it is a relatively large change in composition then effectively it is a different variable at each time-point. P3, Paragraph 2. So a different set of indicators is collected to make up the PMAQ at each round?  Thank you for this important comment. There are three aspects to our response, but essentially this point is very well taken.  First, we have revised the paper to provide more detail on the PMAQ score and, in particular, references of government documents that explain (in painstaking detail) all the indicators that go into the index and how the index is constructed (Methods, paragraph 2 and 3). We also give a breakdown of the indicators by type of indicator, which we believe is informative (Methods, paragraph 2).  Second, we now provide results from a sensitivity analysis in which we develop our own measure of structural quality of care that is based on a common set of indicators from each PMAQ round. The indicators that form this index capture the availability of 92 drugs, 23 items of equipment, and 22 consumables and diagnostic tests. The index is the percentage of items that were present on the day of the external assessment visit. While this index deals with the issue of changes in measurement, the index only measures structural quality and not processes of care, outcomes, and management practices. This is relevant because we would expect bonuses to health workers to be more influential in improving processes of care and management practices since they are more under the control of family health teams (indeed the smaller coefficients for the structural quality of care index are consistent with this statement). We therefore regard the sensitivity analysis as a partial solution to this comment. We have revised the Methods (Statistical Analysis, paragraph 5) and the Results (paragraph 6), and include a new table of results in the appendix (Table S5). As an important aside, our structural quality of care index of 137 items are structural indicators that were measured in exactly the same way over time. This index should not be seen to imply that all the other (non-structural) indicators in the PMAQ score substantially changed over time. Many of the changes to indicators were in fact tweaks to the phrasing of a question rather than changes to what was fundamentally being measured.  Third, while the PMAQ score is measured differently at the two time points, our empirical strategy helps to mitigate this concern. Because the change in the measurement of the PMAQ score is applied to all units (i.e. municipalities), the difference-in-differences approach takes care of this issue (by controlling for time). Given this, it is not easy to see how the results could be an artefact of changes in measurement. The specific indicators chosen in the third round of PMAQ and the method to aggregate them into an overall index would need to have been systematically advantageous for the bonus municipalities relative to the non-bonus municipalities. This is especially difficult to imagine given how many indicators and types of indicator across a broad spectrum of care were used in the construction of the PMAQ score. A final point – which we appreciate does not speak to the question of measurement – is that the PMAQ score was the Government of Brazil’s official metric of quality used within the programme. It therefore carries legitimacy amongst policymakers in Brazil. |
| R1 | 3 | Would each municipality use the same rules to determine how much bonus each team/team member would receive? I.e. is the bonus a universal scheme for team members?  This is a good question but one we cannot comprehensively answer quantitatively. First, a clarification: bonuses were not universal, as less than half of the municipalities gave any bonuses to family health team members.  What we know from the survey is whether municipalities gave health worker bonuses from the PMAQ funds and, if yes, how much on average as a proportion of health worker salaries. We also know from the survey that, amongst the municipalities giving bonuses, around 80% based the amount given to family health team workers on the performance categories determined by PMAQ (through the external evaluation). Unfortunately, we do not have further information on the locally developed rules to determine bonus size. As part of the broader project, we conducted in-depth interviews in around 20 municipalities. These data confirm that municipalities almost universally used the performance score categories to determine how to allocate funds to teams; however, there was variation across municipalities in which workers were eligible for payment and whether bonus amount differed by cadre. |
| R1 | 4 | P4. Paragraph 1. From the study flow diagrams, it looks like step three was unable to be done for some municipalities. Was this due to gaps in the census data (i.e. data not available for a particular census area?), or were some municipalities unable to be allocated to a census area?  In the third step, some family health teams are lost for two possible reasons: 1) there was no census income data for the family health team because of missing geographical information to link it to the census area (not because of missing census area data); 2) there were missing data on the characteristics of the health facility (out of which the family health team operated). We have clarified this point in the caption of the study flow diagram (S2 Figure and S3 Figure). |
| R1 | 5 | P5, Paragraph 1. What method was used to adjust the SEs for clustering at the municipality level?  We the adjust the SEs for clustering because assignment (i.e. exposure) is at the municipality level. We did this in the standard way. In Stata, we used regress and applied the clustered sandwich estimator using the syntax: vce(cluster municipality_code). We have revised the paper (Methods, Statistical Analysis, paragraph 1). |
| R1 | 6 | P5, Paragraph 3. The direct comparison is ok, but I would also consider adding standardised differences for the variables before/after matching as well as this make the comparison easier.  Thank you for this suggestion. We have added such a table to the supporting information, in which we report standardised % bias amongst other statistics (S3 Table). Incorporating these results into Table 1 would make it too busy.  The other check that would be useful to see would be something to demonstrate the degree of common support between the two groups. The last time I used PS matching in Stata (a long time ago though!) the add-on psmatch2 had some basic visualisations for distribution of the propensity score (i.e. an overlapping density plot by group on the same graph) which were effective that should be included in the appendix for the main analysis. There might even be something prettier now.  Thank you for this suggestion. We have added such a figure to the supporting information (Fig S1). |
| R1 | 7 | P5, Paragraph 4. The stratified analysis approach will work - but usually the better way to do this is to include an interaction of the treatment effect with the sub-group variables in the regression model and use an appropriate command/syntax (i.e. margins in Stata) to get treatment effect estimates for each sub-group combination. If only the key variables (i.e. time, treatment group) are included then a stratified approach will effectively the same estimate. If you have included the variables from the propensity score matching process into the final D-in-D model (i.e the 'double-robust approach') then doing a stratified analysis is effectively like including an interaction term of the sub-groups with these variables as well which may not be what was intended. The benefit of the interaction approach is that you can get a p-value for the interaction indicating the level of evidence there is for a difference in treatment effect according to the sub-groups (a more direct approach than comparing 95% CIs which is an approach that is less powerful than the p-value from the interaction).  Thank you for this suggestion. We were aware that doing a stratified analysis is equivalent to including interactions between subgroups and the covariates. And this is not the same as running one regression in which the exposure variable(s) is interacted with the subgroups when covariates are involved.  On balance, we agree with the statistical reviewer and we now follow the more commonly used method of including interactions. There remains though the question of presentation – whether to present subgroup effects as the difference between one group and a reference group, or to show point estimates of each subgroup. We believe Figure 2 had advantages in terms of communicating the findings and we have retained the same format to present the revised results. We agree though that showing the subgroup differences and their p-values is of interest and have inserted a new table into the supporting information. We have revised the Methods (Statistical Analysis, paragraph 4), Figure 2, and added Table S4 in the supporting information. |
| R1 | 8 | P5. Paragraph 5. I am clear about sensitivity analyses except for weighting by population - is this to get results that are interpretable at a population level?  Yes, we wanted to generate estimates that could be interpreted as the average treatment effect for an individual in the population, rather than a family health team. The idea being to give greater weight to family health teams serving larger populations. On reflection, this interpretation is not straight forward (population size is not equivalent to patients using services and the weighting does not account for the fact that there may be multiple family health teams in a census area). For this reason, we have dropped this sensitivity analysis. |
| R1 | 9 | P6. Table 1. I wasn't clear if the matching was 1:1 and without replacement, how the matched sample ended up with more teams who received any bonus than those with no bonus? i.e. shouldn't it be 5052 teams in both groups in the matched sample?  The matching was applied to municipalities, not family health teams. This is why the number of municipalities in each arm were the same. The number of teams in a given municipality varies. We have clarified in the paper that the matching is done at the municipality level (Methods, Statistical Analysis, paragraph 3). |
| R1 | 10 | Fig 1. I'd recommend to use an alternative plot to the bar plot with CI lines - depending on the size of the dataset in each of the bonus categories, a boxplot with jittered points, or if too much data then a plot that shows the distribution would be much more useful, i.e. a 'violin plot'.  We appreciate the desire for more information on the distribution of the data with this suggestion. We have added a new figure in the supporting information showing violin plots (S4a Fig and S4b Fig). We have retained Figure 1 because we believe it communicates well the changes over time in the different exposure groups (while noting that even this figure is not straight forward to read with 10 different groups). The violin plots, while useful in showing distributions, are very busy and do not communicate well the key messages of Figure 1. |
| R1 | 11 | Fig 2. I would clarify this as the 'income of team members' in the caption.  We have clarified in the caption this is the average monthly income of households in the local area of the family health team (Figure 2 caption). |

## Reviewer 2

| R2 | 1 | Thank you for sharing this interesting paper on a major P4P programme in Brazil. The work is very well written and expertly carried out. The writing is clear and understandable, and the methods are appropriate. The inclusion of multiple sensitivity analyses is welcome and these demonstrate the robustness of the work. I have no major comments or changes suggested. |
| --- | --- | --- |
| R2 | 2 | Clarify early on (in abstract) that this study is about evaluating rewarding health professionals, as the term "family health teams" could imply service providers or their budgets.  Thank you for your comment. This was an unintentional error on our part. We have now revised the introduction part of the abstract to clarify that the study is about giving bonuses to family health team workers, not family health teams (Abstract). |
| R2 | 3 | - I think it would be useful to mention the size of the overall PMAQ as proportion of public spending - I believe it was relatively small.  Thank you for your comment. We have revised the paper to include this information (Methods, paragraph). PMAQ accounted for approximately 10% of federal expenditure on primary health care over the period 2014-2017. |
| R2 | 4 | - Would it be worth mentioning in the limitations that municipalities that give incentives to teams could be different in characteristics that were not measured (political factors, working arrangements, local contracting, existing salaries and other bonuses) and could have had some minor bias;  Thank you for this comment. This is indeed true, although we would clarify that these unmeasured confounders would need to be time-varying to be a threat. In the revised paper, we now mention those factors suggested by the reviewer as potential confounders (Discussion, paragraph 6). |
| R2 | 5 | - I would emphasize more the uncertainty about clinical relevance of the PMAQ surveys and scores;  This point is well taken. We now mention this in the abstract as a limitation to provide more emphasis (Abstract). |
| R2 | 6 | - Could the authors also comment on the size of the bonus - 21% or 50%+ of salary - where most of the impact was found. I think this is a relatively large amount, and do they recommend this approach be used in other settings for relatively modest quality gains?  This is a good question, without a straight forward answer. We want to reiterate that the findings need careful interpretation. We are not showing the impact of giving large bonuses relative to the absence of any funding to family health teams. If that were the interpretation – or rather the study design – it certainly would be questionable whether giving large bonuses are “worth” the relatively modest quality gains reported. However, given the study design, we are showing whether giving large bonuses to health workers instead of purely input-based financing is associated with quality gains. On the face of it, and with various caveats given in the Discussion already, the findings would suggest this approach is to be recommended. From a policy perspective, however, there are other considerations. For example, how sustainable are the bonuses to the health workers, and what would be the consequences for motivation and quality of care if they were to be withdrawn at a later date? These are tricky questions and lengthy speculation is arguably beyond the scope of our paper. Nonetheless, we have revised the paper to at least raise these important issues (Discussion, para 8). |

## Reviewer 3

| R3 | 1 | Reviewer #3: Paper studies "a national health financing programme to improve access to and quality of primary health care" (PMAQ) in Brazil. Through this program, "the federal government made financial payments to municipalities based on the performance of family health teams. […] Municipalities had the flexibility to decide whether to retain payments at the municipal level or redirect them to the family health team level, […] could decide how to use the financial resources which means we can compare municipalities that gave bonuses to health workers with those that chose to invest solely in the supply-side readiness of the primary care system. Finally, municipalities differed in the size of bonus paid to family health teams."  From this initial description, the reviewer is sceptical about the authors claim that "PMAQ provides an ideal testing ground for addressing three questions about scheme design …," because much of the design features are the consequence of decisions taken at the municipal level, the classification as a "quasi-experimental" study is therefore misleading, as is the statement that the study "exploited municipality variation in the design features" of the national program. Instead, the design of PMAQ appears uniform across municipalities; within this framework, the study addresses choices made to best effect by municipal authorities.  We apologise if we have not fully understood this comment. Based on our understanding, we believe there is no substantive difference of opinion here. Before giving our response, it is important to make clear that we are not evaluating PMAQ, and indeed we emphasised this point in the Discussion.  PMAQ was a national programme. Some of the design features were national in scope and were applied across all municipalities in a uniform way. However, crucially for our study, the federal level gave municipalities discretion on various aspect of scheme design. The most notable feature was how the PMAQ funds could be used – i.e. to give as bonuses to health workers or as input-based financing. We exploit this variation in our study, not to evaluate PMAQ per se, but to provide evidence on whether performance bonuses to health workers are associated with better quality of care compared with more traditional input-based financing. PMAQ thus provides the context allowing us to address our research question. We agree that “the study addresses choices made by municipal authorities”. These choices are choices that relate to P4P scheme design. To improve clarity, we have revised the Introduction to emphasise the design features that were determined at different levels (Introduction, paragraph 3).  Please also note that we have revised the title in line with the comments of the Editor. We no longer refer to quasi-experimental and instead label the study as a difference-in-differences analysis – which we agree is a more precise and informative description of the study design. Still, the difference-in-differences approach is one of the most common quasi-experimental methods used in the medical and social sciences, and we do not believe this umbrella term is misleading.  We apologise but we do not understand the objection to the phrase “ideal testing ground”. We defer to the Editor – we are happy to remove the word “ideal” and replace it with a different adjective if requested. |
| --- | --- | --- |
| R3 | 2 | The study adopts a difference-in-difference approach, comparing changes in outcomes (a composite indicator on health service provision collected under PMAQ) across municipalities adopting bonuses vs those who spent it on supply-side strengthening. Potential bias from self-selection of municipal authorities into arrangements on how the funds are used is addressed by a procedure matching each of the "bonus" municipalities to a control. The methods and their motivation are explained adequately though somewhat generically. Some important aspects of the methods are left unclear: (1) What is the "full set of baseline municipality, facility and local area characteristics" the study controls for?  Thank you for your comment. We have now clarified what matching variables we used (Methods, Statistical Analysis, paragraph 3). |
| R3 | 3 | (2) The matching procedure, a crucial aspect of the study as it potentially mitigates the bias inherent arising from the endogeneity of the design decisions on the municipal level, is documented rather parsimoniously (considering it is crucial in relation to the objectives of the study), largely indirectly in connection with the data description under results (Table 1).  We have now clarified what the matching variables are, so we presume this comment is referring to the one-to-one matching procedure. We defer to the Editor whether we need to provide more details since one-to-one matching is standard in the literature. We also note that the statistical reviewer did not comment on this point. Essentially, the procedure is as follows. A treatment unit finds the control unit with the nearest propensity score. If the difference in propensity score falls within the caliper, the match is successful and the control unit is taken out of the sample (i.e. without replacement). The same procedure is applied to the next treatment unit, and so on. Control units that are never matched are discarded. Treatment units that never find a match are discarded. |
| R3 | 4 | (3) Relatedly, the final step in the process by which the sample is whittled down to 2346 municipalities through the matching procedure is opaque.  The sample is reduced down from 3371 to 2346 municipalities because of the one-to-one matching procedure, as explained in the text and in our response to the previous comment. |
| R3 | 5 | Result tables are presented and summarized competently. However, two figures referred to in the text are missing from the paper.  Our apologies. These figures were in the supporting information. Our understanding is that the submission guidelines gave this as an option. |
| R3 | 6 | The discussion of shortcomings of the study is competent, making pointers to the issues raised in this review. E.g., it notes that "our study design cannot rule out unmeasured confounding and is therefore unable to provide definitive evidence of a causal relationship." |
| R3 | 7 | Overall, this is a competent study. The style and presentation are crisp and language is consistently of high standard. However, the framing of the study as an ideal setting for a DiD approach is not supported by the data characteristics - the fact that the decisions on the design features the study focuses on are made on the municipal level is highly problematic for the purposes of the analysis. This - and the sensible steps the authors have taken to mitigate this problem - needs to be communicated more clearly. Editorial note: Consider adding a digit to some estimated coefficients which are small in absolute numbers (e.g., "0.0" for population size or clinical staff).  In the paper, we stated that PMAQ provided an ideal testing ground for addressing certain questions on P4P scheme design. We did not use language characterising the “study as an ideal setting for a DiD approach”.  Whether our study design – difference-in-differences with matching – is a credible approach to estimate the effect of bonuses to health workers is of course an important question. The study remains vulnerable to unobserved time-varying confounders, even if we tried to address some of these through the matching procedure. We performed various sensitivity analyses to provide supportive evidence that our results are robust.  We have made several changes to the paper that relate to this comment. First, throughout the paper we refer to associations, instead of effects (see our response to the Editor). Second, we have elaborated on aspects of the Methods to provide greater clarity (see our responses to previous comments). |

## Reviewer 4

| R4 | 1 | Reviewer #4: This is a very nice written report on assessment of a large pay-for-performance (P4P) programme (called PMAQ) in Brazil Federal 5000 municipalities to improve primary health care quality. The authros used a quasi-experimental design differences-in-differes with matching (and without matching). They took leverage fact that the money was sent to the municipal level which decided how to use (from improving infrastructure to incentivise the health workers/health workers family teams). These actions where quite variable to build different forms of the exposure variable: either binary (any bonus to family health teams vs none) and multinominal (levels of the size of bonus). They found statistically significant association (apparentely U-shaped) robust to many sensitivity analyses. |
| --- | --- | --- |
| R4 | 2 | It would be good to have further details on how this PMAQ score (which varies from 0 to 100) is built. - Please add further details on this.  Thank you for this comment. Since several reviewers have raised this issue (see our response to R1.2), we have added further details in the Methods (paragraph 2 and 3) at the risk of repeating details contained within another paper we published on PMAQ. |
| R4 | 3 | - The authors use linear regression to analyse the change of the PMAQ score (as continuous variable) however none knows what that means in substantive terms (the validity issue the authors point oult in the limitation) (eg: is a change of 1 the same as in 10 unities). This makes quite hard to appreciate in substantive terms the statistically significant results presented.  Thank you for this comment. It reflects the challenge of interpreting results from a composite index. One way we tried to address such a comment in our original submission was to report estimates in relative terms. For example, the main estimate suggests bonuses are associated with a 7.5% relative improvement in the PMAQ score. This helps in giving some sense of the magnitude. In the Discussion we then reported findings from other P4P studies, to provide some benchmarks and again to help with interpretation of magnitudes.  An additional approach could be to report the standard deviation of the PMAQ score in round three when describing the results from Table 2. For example, the SD of the PMAQ score in round three is 12.6 points in the matched sample. So the estimate (for the association of bonus payments with quality) of 4.6 points is roughly 0.4 of a SD. However, we are not convinced this would help readers and may even confuse matters. We are happy to do this if the reviewer and Editor believe this will be helpful.  Please note that we have changed the paper to report absolute estimates in terms of “points” rather than “percentage points”. We feel this is technically more correct, even if the score runs from 0 to 100. |
| R4 | 4 | - Please provide more descriptive statistics of PMAQ round 1 (eg median and IQR on table 1) and as well as for PMAQ 3 and the change of the score. Had you done deciles of PMAQ round 1 how many would improved their deciles for example? This is OK to be as supplementary materials.  In response to the statistical reviewer, we now show the distribution of the PMAQ score by round and exposure group using violin plots. These are reported in the supplementary material. We believe this addresses this comment – which is asking for statistics that reveal something about the distribution of the outcome variable. |
| R4 | 4 | Table 1: the total population in what unities is this?  Apologies this was not clear. The variable is population / 100,000. We have made this clear in caption of Table 1. |
| R4 | 5 | Table 2 and similar tables in the supplementary materials: please add the reference category for the binary and categorical variables (PMAQ bonus, PMAQ bonus size, Local area and health facility type)  In the caption to Table 2 and other tables in the supplementary materials, we have added a note indicating the reference categories. |
