# Supplementary material for: Performance bonuses and the quality of primary health care delivered by family health teams in Brazil: A difference-in-differences analysis
Source: PLoS Med. 2022 Jul 7;19(7):e1004033. doi: 10.1371/journal.pmed.1004033 (PMC9262241; doi:10.1371/journal.pmed.1004033)
Supplement: Attachment — Submitted filename: Responses_RRR.docx [file pmed.1004033.s019.docx]

Thank you for these additional comments. Please see our responses below in blue (we have only included the comments that implied a response is needed).

**Requests from Editors:**

1. Please include line numbers in your next draft

We have done this.

2. In the abstract and main text please avoid implications of causality (“... increase in the PMAQ score”; “positive impact”, “a more effective way”); however, we kindly request that you use more restrained language in describing findings, e.g. “associations” ,“evidence of an apparent benefit …”or similar

We have done this.

3. Regarding data availability, thank you for providing an appropriate contact for inquiries on restricted data. This will suffice for researchers who may wish to obtain the full data set for PMAQ score variables and exposure variables to replicate analyses.

No response required.

4. For the restricted data, please provide a “minimal data set” which consists of the data set used to reach the conclusions drawn in the manuscript with related metadata and methods, and any additional data required to replicate the reported study findings in their entirety. Authors do not need to submit their entire data set, or the raw data collected during an investigation. Please submit the following data:
   a) The values behind the means, standard deviations and other measures reported;
   b) The values used to build graphs;
   c) The points extracted from images for analysis

By email, we previously followed up with some questions in regard to data availability and look forward to a response. Here, we have an additional query, as we are not quite sure what is meant by “the values behind the means, standard deviations and other measures reported”? Grateful for more explanation.

We now provide the raw data in response to 3b and 3c above.

5. Introduction - first sentence of the last paragraph should read “mattered in Brazil,” and not “mattered. in Brazil.”

We have done this.

6. In your tables (e.g. S6, S7) please do not report P<0.0001; report as P < 0.001

We have done this.

7. References – ref #38 seems incomplete. Please include the journal details

We have removed this reference and now cite Basinga et al (which was already in the reference list)

8. Please remove the “Data availability” statement at the end of the main text. This information is captured in the metadata obtained in the submission form

We have done this.

**Reviewer #1:**

9. Abstract, Methods and finding: I'd say '…least square regression' instead of the plural

We have done this.

10. For the sub-group analyses, to clarify, the p-value I referred to in the earlier review was the overall test of including the interaction - you could do this with lrtest "interaction*term" in Stata that provides an overall test the model with the interaction included vs. a model without it. The individual p-values for each marginal effect aren't really needed, and you could just remove these and add the overall p-value for each sub-group section (e.g. 1 p-value for PMAQ bonus).

We perhaps follow what the reviewer is suggesting here, but we are not completely confident. To avoid misinterpreting this suggestion, we haven’t made any changes in response. Moreover, we think the current approach of presenting individual p-values for each marginal effect is not incorrect, even if it is perhaps over-kill.
