# Supplementary material for: Performance bonuses and the quality of primary health care delivered by family health teams in Brazil: A difference-in-differences analysis
Source: PLoS Med. 2022 Jul 7;19(7):e1004033. doi: 10.1371/journal.pmed.1004033 (PMC9262241; doi:10.1371/journal.pmed.1004033)
Supplement: Attachment — Submitted filename: Responses_RRRR.docx [file pmed.1004033.s020.docx]

Thank you for these additional comments. Please see our responses below in blue (we have only included the comments that implied a response is needed).

1. Discussion line #347, should read “… overall impact on PMAQ” and not “…overall impact of PMAQ)

This entire sentence was in fact not very clearly (which is probably what motivated this comment). We have revised the text to say “It is important to emphasise that we did not evaluate PMAQ, and the results should not be interpreted as estimates of the impact of the programme.”

2. Discussion line # 387 is missing a word. It should read “..the availability of a..”

We have done this.

3. I think there's just one adjust to make, and it is regarding the p-values for each marginal effect. I would agree that overall there are general consistent effects across the subgroups, the issue is that comparing p-values between levels of a sub-group variable is a phenomenon called 'differences in nominal significance', where the bonus * subgroup effect is checked by comparing the whether the p-value for one subgroup is above/below the threshold vs. another p-value. There are good reasons not to do this (it often leads to the wrong inference, e.g. in an extreme situation where one subgroup level has p=0.051 and another p=0.049). The most powerful approach to testing whether or not there are consistent effects of payment according to PMAQ/municipality characteristics is a likelihood-ratio test of all parameters associated with the interaction between bonus and subgroup..

We think we now understand the substance of this comment and agree with it. Our understanding is that a likelihood-ratio test is done after a logit (or similar) model. We have therefore done something similar for an OLS model, by performing a Wald test that the income subgroup effects (by which we mean the subgroup differences using the poorest group as the reference category, as reported in S4 Table) are jointly equal to zero. This produces a single p value for this joint test of whether there is heterogeneity, and is equivalent we believe to what the reviewer was suggesting. The changes are reflected in a new version of S4 Table.
